# Measuring bullying victimization through a closed ended question and a validated measure in a population of tunisian adolescents: What difference does it make?

**DOI:** 10.1192/j.eurpsy.2021.575

**Published:** 2021-08-13

**Authors:** N. Brigui, A. Guedria, T. Brahim, R. Ayoub

**Affiliations:** 1 Child And Adolescent Psychiatry Department, Fattouma Bourguiba University Hospital, Monastir, Tunisia; 2 Research Laboratory, R05es10, Faculty Of Medicine Of Monastir, Tunisia, Psychiatry Department, Fattouma Bourguiba University Hospital, monastir, Tunisia

**Keywords:** adolescent, bullying, measurment, prevalence

## Abstract

**Introduction:**

School bullying is a serious problem among tunisian children and adolesent. In our every day practice, we see a considerable number of suicide attempts among bullying victims. Our study tries to provide more information on this phenomenon, in order to organize efficient preventtion measures.

**Objectives:**

Measuring the prevalence of bullying victimization in the town of Sousse-Tunisia Comparing the prevalence found through a validated measurement tool and a closed end question

**Methods:**

It is a cross-sectional study among a sample of 1127 adolescents, aged 12 to 17 years old. The adolescents were divided in two groups the first group, composed of 527 adolescents, answered a closed end (yes/no) question “Have you been a victim of Bullying”; the second group, composed of 600 adolescents, responded to the “adolescent peer relation instrument”.

**Results:**

The first group was composed of 48% of boys and 52% of girls with a mean age of 13,24 ± 0,96. The second group was composed of 50% of boys and 50% of girls with a mean age of 13.76 ±1.37. We found a bullying victimization prevalence of 11% for the first group versus 95.1% for the second group. For both groups we didn’t find a significant difference in the prevalence of bullying victimization according to sociodemographic factors except the higher family income that was associates to less bullying victimisation for the first group (p=0,04)

**Conclusions:**

The high prevalence of bullying victimization we found using a validated measurement is alarming in terms of the urgency of interventions to prevent bullying in schools.

